# Current Evidence and Intervention Approaches for Physical Activity and Low Back Pain in Older Adult Populations: A Narrative Review

**DOI:** 10.1155/bmri/2625502

**Published:** 2026-06-11

**Authors:** Walid Kamal Abdelbasset, Tamer M. Shousha, Gopal Nambi, Alshimaa R. Azab, Ahmed M. Abodonya, Fatimah W. Abdelrahman

**Affiliations:** ^1^ Department of Physiotherapy, College of Health Sciences, University of Sharjah, Sharjah, UAE, sharjah.ac.ae; ^2^ Department of Health and Rehabilitation Sciences, College of Applied Medical Sciences, Prince Sattam bin Abdulaziz University, Al-Kharj, Saudi Arabia, psau.edu.sa; ^3^ Department of Surgery, College of Medicine, Prince Sattam bin Abdulaziz University, Al-Kharj, Saudi Arabia, medcol.mw

**Keywords:** elderly, exercise, low back pain, physical activity, rehabilitation

## Abstract

Low back pain (LBP) is a common and severe musculoskeletal ailment that disproportionately affects the elderly. Some of the top causes of disability and impaired functional independence occur worldwide. As populations age and chronic diseases increase, controlling LBP in the elderly is a public health issue. Polypharmacy and age‐related vulnerabilities make widely recommended pharmacologic methods risky for older persons. Physical activity is a favored first‐line remedy due to its safety and multidimensional advantages, especially in the elderly. This review synthesizes the literature on elderly LBP prevention and management through physical exercise. Recent evidence (2020–2024), biological underpinnings of exercise‐related pain regulation, and practical applications of aerobic, resistance, flexibility, and neuromuscular training are highlighted. The biopsychosocial and Kinesio pathophysiological frameworks that inform LBP rehabilitation therapeutic thinking are also reviewed. Fear of pain, social isolation, comorbidities, and environmental limits are examined, along with ways to increase exercise engagement and adherence. This article also assesses literature discrepancies, identifies study gaps, and suggests further research. To help healthcare providers and researchers optimize exercise‐based interventions for the elderly. Physical activity should be a standard therapy method for senior LBP patients, not an optional recommendation.

## 1. Introduction

Low back pain (LBP) is a major contributor to disability, especially in community‐dwelling older adults. The World Health Organization and Global Burden of Disease Study have consistently ranked LBP as the leading cause of years lived with disability globally [1]. In this review, the term “older adults” refers to individuals aged 65 years and above, consistent with commonly used geriatric age thresholds in the literature [2].

In order to successfully personalize therapies, it is necessary to classify the heterogeneity of LBP in older persons. The majority of LBP cases (85%–90%) are classified as nonspecific because they do not have a clear cause, such as nerve compression or stenosis. Instead, they result from a combination of factors, such as imbalances in the muscles, degeneration, and central sensitization. Lumbar radiculopathy, which can be caused by disc herniation or foraminal stenosis, is known as radiating leg discomfort, sensory alterations, or weakness, as demonstrated by a positive straight leg raise test, and is a symptom of radicular LBP. Neurogenic claudication, which is characterized by bilateral leg pain, paralysis, or numbness alleviated by flexion, is caused by lumbar spinal stenosis (LSS), which affects 10%–20% of individuals over the age of 70 [2–5].

In elderly populations, LBP often coexists with other chronic illnesses hindering mobility, independence, and quality of life. According to recent surveys, approximately 36%–70% of individuals aged 65 and older experience LBP, with a significant proportion reporting persistent or recurrent symptoms [5]. Hence, the consequences of chronic LBP in the elderly are multifaceted, including loss of autonomy, risk of falls, and higher healthcare utilization. These outcomes also contribute to emotional distress, social isolation, and economic burden on both patients and healthcare systems. Given the growing demographic shift toward an aging society, effective management strategies for LBP in older adults are urgently needed [6].

Pharmacologic treatment, including analgesics, NSAIDs, and muscle relaxants, remains the most frequently prescribed first‐line intervention for elderly patients with back pain. However, this approach has its disadvantages. For instance, polypharmacy, a common issue in geriatric medicine, increases the potential for adverse drug reactions. NSAIDs, for example, can lead to gastrointestinal bleeding and renal impairment, whereas opioids may result in sedation, cognitive impairment, and falls [5, 6]. Moreover, surgical interventions are effective in certain cases; however, they carry significant perioperative risks for older adults, including delayed recovery, infection, and anesthesia complications. As such, nonpharmacological alternatives that are both effective and safe are increasingly being prioritized in clinical guidelines and public health strategies [7].

Therefore, physical activity encompasses all movement produced by skeletal muscles resulting in energy expenditure and includes structured exercise, recreational movement, and daily physical tasks. Over the past two decades, exercise has evolved from being viewed as a supplementary recommendation to becoming a foundational element of musculoskeletal rehabilitation. Its benefits extend beyond musculoskeletal health, encompassing cardiovascular fitness, cognitive function, and emotional well‐being [8]. In the context of LBP, physical activity is particularly effective in addressing the multifactorial nature of pain. It not only strengthens muscles that support the spine but also modifies pain‐processing pathways and enhances psychological resilience. Numerous studies have confirmed that exercise reduces pain severity, improves functional outcomes, and enhances quality of life among older adults with LBP [9, 10].

The purpose of this review is to critically evaluate recent literature (2020–2024) on physical activity as a modality for preventing and treating LBP in elderly individuals. This narrative review aims to: contextualize current findings within theoretical models such as biopsychosocial, Kinesio pathological; discuss the physiological and psychological mechanisms by which physical activity impacts LBP; explore specific types of exercise (aerobic, resistance, balance, flexibility, and mind‐body); identify barriers to participation and strategies to enhance adherence; examine opposing viewpoints and limitations within the current evidence base; and lastly, provide clinical and public health recommendations.

## 2. Theoretical Frameworks for Understanding LBP in the Elderly

Understanding the mechanisms and experience of LBP in older adults requires an interdisciplinary perspective. Two principal theoretical models dominate the literature: the biopsychosocial model and the Kinesio pathological model. These frameworks provide valuable insights into how physical activity can serve as a multifaceted intervention for LBP. It was reported that the biopsychosocial model posits that chronic pain, including LBP, arises not solely from tissue damage but from an interaction between biological, psychological, and social factors. This model has become the dominant paradigm for explaining persistent pain and disability, especially in the elderly [11]. First, biological factors relevant to LBP include age‐related structural changes, such as intervertebral disc degeneration, osteoarthritis of the facet joints, and decreased paraspinal muscle mass [12]. Second, psychological factors encompass fear of movement, pain catastrophizing, and depression elements, which are common in elderly individuals coping with chronic illness and disability. Social factors may include isolation, lack of support, and socioeconomic constraints [13].

Physical activity influences each of these domains. For example, aerobic exercise can decrease inflammation and improve sleep; resistance training strengthens muscles and enhances function; group‐based or socially supported exercise improves mood and motivation [10, 14]. Interventions guided by the biopsychosocial model recognize that exercise is not just about restoring physical function but also about modifying perceptions of pain, improving mood, and increasing social interaction [13]. A recent study by de Roode et al. [7] confirmed that context‐driven, patient‐centered exercise programs rooted in the biopsychosocial model resulted in significantly better pain relief and functional improvement than traditional “exercise‐only” models that ignored individual psychosocial circumstances [8].

The kinesiopathological model, which emphasizes movement system dysfunction, views chronic LBP because of recurrent, direction‐specific movement and alignment errors that eventually overload spinal tissues, in contrast to the biopsychosocial model. Repetitive lumbar flexion or extension, relative stiffness between lumbar and hip segments, and poor muscle recruitment (such as delayed multifidus or transversus abdominis activation) can cause microtrauma and discomfort. Sarcopenia, spinal mobility loss, and decreased proprioception enhance these movement defects in older persons, making habitual postures (prolonged sitting, stooped walking) and ADL compensatory strategies primary causes of mechanical LBP [15, 16].

When seen through this lens, exercise serves as a tool for conditioning as well as a method for achieving more even distribution of body weight and movement patterns throughout the spine, pelvis, and hips. To improve lumbopelvic rhythm and restore segmental control, dynamic stabilization, focused lumbar extensor strengthening, and motor control exercises are used. On the other hand, task‐specific training, such as sit‐to‐stand, walking, and stair climbing, focuses on correcting poor strategies seen in functional activities. To better align the kinesiopathological model with broader geriatric rehabilitation goals, it is especially relevant to incorporate balance and postural control exercises such as Tai Chi and trunk stability work into the treatment of the elderly. These interventions enhance the quality of movement while reducing the risk of falls [16–18].

A kinesiopathological model attributes pain to specific mechanical faults like repetitive lumbar extension or hip‐lumbar stiffness causing tissue overload, which can be corrected through targeted retraining. On the other hand, the biopsychosocial model explains elderly LBP through interactive biological (degeneration), psychological (fear‐avoidance), and social (isolation) factors, with physical activity addressing all three holistically. When used in conjunction with a biopsychosocial framework, such as cognitive functional therapy, MSI allows for more accurate biological targeting such as exercise programs tailored to each individual′s needs and greater results in geriatric rehabilitation than either approach alone [14, 16, 18].

By describing how alterations brought on by aging exacerbate movement errors that put an excessive strain on tissues, the kinesiopathological (MSI) model establishes a connection between musculoskeletal deterioration and LBP. As a result of sarcopenia, paraspinal muscular mass and strength decrease (20%–40% loss by age 70), which hinders recruitment of the multifidus and transversus abdominis and segmental stability, ultimately leading to compensatory overuse of the lumbar region. When spinal discs and facet joints deteriorate, movement becomes more restricted, leading to stiff postures (such as thoracic kyphosis and an enlarged sagittal vertical axis) and higher strain on the spine as a result of flexion and extension. Lumbar dominance and hip stiffness are two examples of how proprioceptive loss (few robust muscle spindles and afferent pathways) exacerbate lumbopelvic rhythm [17–19].

Precisive decline (reduced muscle spindle sensitivity, afferent degeneration) worsens lumbar position sensing and lumbopelvic coordination, amplifies the effects of age‐related physiological changes, and increases reliance on vision and spinal overload, all of which exacerbate MSI defects. Reduced strength in the lower extremities causes proprioceptive weighting to shift to the trunk, which in turn increases the risk of falling while standing or walking and increases the swaying of the center of gravity. By the 70s, Type II atrophy accounts for 20%–40% of muscle fiber turnover, which slows recruitment and promotes persistent low‐load postures that perpetuate overload. Satellite cell failure also hinders healing. Resistance training, neuromuscular activation, and muscle hypertrophy all work together to counteract these effects [19–22].

Precisive decline (reduced muscle spindle sensitivity, afferent degeneration) worsens lumbar position sensing and lumbopelvic coordination, amplifies the effects of age‐related physiological changes, and increases reliance on vision and spinal overload, all of which exacerbate MSI defects. Reduced strength in the lower extremities causes proprioceptive weighting to shift to the trunk, which in turn increases the risk of falling while standing or walking and increases the swaying of the center of gravity. By the 70s, type II atrophy accounts for 20%–40% of muscle fiber turnover, which slows recruitment and promotes persistent low‐load postures that perpetuate overload. Satellite cell failure also hinders healing. Resistance training, neuromuscular activation, and muscle hypertrophy all work together to counteract these effects [19, 21].

Degenerative changes worsen MSI faults in the elderly. Sarcopenia reduces the endurance of the lumbar extensors and deep stabilizer activation, disc height loss stiffens the motion segments, and proprioceptive deficits disrupt movement coordination due to muscle spindle degeneration and central processing decline. A vicious cycle where deterioration worsens deficits and vice versa is created by habitual forward leaning (kyphosis) or sedentary postures, which burden the lumbar facets/discs. The restoration of recruitment time, segmental mobility, and postural habits that occur after exercise disturbs this [4, 23].

Rather than viewing these models as mutually exclusive, many modern rehabilitation strategies seek to integrate them. For example, motor control exercises informed by Kinesio pathological principles can be delivered within a biopsychosocial framework, which also addresses fear‐avoidance, self‐efficacy, and participation in life roles [24]. Utilizing both models can help establish a detailed framework for clinicians to utilize for better patient‐centered care.

### 2.1. Mechanisms of Physical Activity in LBP Relief

The therapeutic benefits of physical activity for LBP in the elderly are underpinned by a series of interconnected physiological, neurological, and psychological mechanisms. Exercise has been shown to reduce systemic inflammation by downregulating proinflammatory cytokines, upregulating anti‐inflammatory mediators like IL‐10, and enhancing muscle metabolism, which reduces adipose‐derived inflammatory signals [25]. Additionally, resistance training improves muscle hypertrophy, fiber recruitment, and neuromuscular activation. Specifically, strengthening the lumbar extensors, gluteal, and abdominals enhances spinal support and reduces mechanical load on vertebral structures [23, 26]. Steele et al. also demonstrated that a progressive resistance training program improved lumbar extensor endurance by 38% in elderly LBP patients, with corresponding reductions in visual analogue scale (VAS) pain scores [27].

Furthermore, postural dysfunction is a key contributor to chronic LBP. Flexed spinal alignment, forward head posture, and pelvic tilt increase spinal compressive forces. Exercise improves kinesthetic awareness, helping elderly individuals adopt safer and more ergonomic movement patterns [28]. Hence, functional training, balance exercises, and proprioceptive drills retrain posture and improve trunk control during walking, stair climbing, and transfers [29].

Central pain processing is major factor that physical activity influences where exercise induces release of endorphins and enkephalins natural opioids that reduce pain perception, enhanced descending inhibitory control, reducing spinal cord hyperexcitability, and reduction in central sensitization, where the nervous system becomes overly responsive to pain stimuli [30]. Therefore, physical activity improves cognitive function, executive planning, and emotional regulation, all of which are often impaired in chronic pain syndromes. Exercise elevates dopamine and serotonin levels, reducing symptoms of depression and anxiety [31].

Regular movement can also reduce fear‐avoidance behaviors, breaking the cycle of pain‐related inactivity and disability. Hence, participation in group‐based programs or supervised sessions provides additional psychosocial benefits, enhancing motivation and accountability. Lastly, sleep disturbances are common in older adults with chronic LBP [32]. Physical activity, especially aerobic and mind‐body practices, promotes deeper sleep, reduces nocturnal awakenings, and improves circadian rhythm stability. Sleep improvements, in turn, reduce pain amplification and increase daytime activity, contributing to a positive feedback loop of recovery and independence [33].

Briefly, there are a number of interrelated biochemical and neuromechanical processes that, when activated, alleviate LBP in the elderly. In order to decrease nociceptor sensitivity in deteriorated spinal tissues, the myokines released by contracting muscles during aerobic and resistance exercise (exercise‐induced IL‐6) downregulate proinflammatory cytokines (TNF‐*α* ↓20%–30%) and upregulate IL‐10. Through dispersing compressive loads from discs and facets and increasing endurance (↑38%), resistance training can reverse sarcopenia by increasing the cross‐sectional area of the lumbar extensors (↑10%–20%). Proper proprioception (a change in joint position of ↓15%–25%) and lumbopelvic rhythm can be achieved by balance and neuromuscular exercises. This, in turn, can alleviate the kyphotic postures that increase strain on the spine [34]. New avenues are opened up by psychosocial variables and central nervous system pain processing. To combat the central sensitization common in chronic elderly LBP, exercise triggers the production of *β*‐endorphin (↑25%–50%) and activates descending inhibition (PAG‐RVM). Group programs improve the odds of adherence in seniors with multiple chronic conditions by increasing self‐efficacy and social support, whereas graded exposure decreases fear‐avoidance behaviors by 30% (TAMPA scale). Benefits from aerobic and mind‐body exercise last longer because they increase sleep quality, which in turn breaks the pain‐vigilance cycles [35, 36].

## 3. Exercise Modalities and Their Evidence Base

A core objective in managing LBP in elderly individuals is to prescribe safe, effective, and personalized physical activity programs. Numerous clinical studies and meta‐analyses have evaluated the efficacy of various exercise modalities. Each modality offers unique benefits based on the individual′s pain profile, physical function, comorbidities, and preferences [37]. This section outlines the most prescribed forms of exercise for elderly adults with LBP, summarizing the evidence base and clinical considerations for each.

### 3.1. Aerobic Training

Aerobic exercise involves rhythmic, sustained movement that improves cardiorespiratory endurance. Common forms include walking, cycling, and aquatic therapy. For elderly adults, walking is often the most accessible and cost‐effective intervention [38]. Pocovi et al. conducted a multicenter randomized controlled trial (RCT) involving 701 older adults with chronic LBP. Participants who walked for at least 30 min three to five times per week experienced a 50% reduction in recurrence of pain episodes over 12 months compared with those receiving standard care. Improvements were also noted in balance and self‐reported disability (measured by the Oswestry Disability Index) [39]. Additionally, a systematic review by Li et al. concluded that low‐impact aerobic exercise was associated with moderate to large improvements in physical function and pain intensity in older adults, with a low risk of adverse effects [40]. It was documented that aerobic activity and core stability exercises improved functional ability, physical performance, fall risk, pain intensity, and depression in LBP patients [41].

### 3.2. Resistance Training

Resistance or strength training involves exercises that enhance muscle force production, which can be performed using body weight, resistance bands, machines, or free weights. In elderly individuals with LBP, the primary focus is on core stabilization, strengthening of spinal extensors, hip abductors, and lower limb musculature, as these regions are commonly weakened and contribute to functional limitations [42].

Steele et al. conducted a RCT in 2021 involving elderly participants (mean age 70) with nonspecific LBP. The intervention group performed supervised lumbar extensor exercises twice weekly for 8 weeks, leading to a 38% improvement in spinal endurance, a 28% reduction in pain scores on the VAS, and significant improvements in walking speed and sit‐to‐stand test performance [27]. Similarly, Zoete et al. compared a combined resistance and aerobic training program to an education‐only intervention. The exercise group demonstrated superior outcomes in functional ability, pain reduction, and mood after 12 weeks, with benefits sustained at 6‐month follow‐up [43].

Clinically, resistance training in elderly individuals with LBP should be approached with attention to safety and adaptation to individual capacity. Low resistance and high repetitions, two sets of 12–15 repetitions are recommended to reduce joint stress and promote endurance [44]. Emphasis should be placed on proper form and technique rather than lifting heavy loads, particularly in populations at risk for injury. Common core‐focused exercises such as bridges, bird–dogs, and seated leg raises are effective in enhancing trunk stability. Additionally, cardiovascular parameters such as blood pressure and heart rate should be monitored during exercise, especially in individuals with known cardiovascular conditions [45].

### 3.3. Flexibility and Stretching Exercises

Flexibility exercises target muscles and soft tissues that may restrict joint mobility and alter normal movement patterns. In elderly adults with LBP, tight hamstrings, hip flexors, and lumbar paraspinal muscles are commonly observed, contributing to postural strain and limited mobility. Regular stretching has been shown to improve range of motion, reduce musculoskeletal stiffness, and alleviate tension in chronically tight areas, ultimately supporting functional independence in older populations [46].

A 2023 systematic review by Zhang et al. examined the effects of various exercise therapies on chronic LBP in the elderly. The study found that exercise interventions, including flexibility training, significantly improved mobility and quality of life while reducing pain and disability [47]. Additionally, Yoga‐based stretching programs have shown additional benefits related to breathing control, balance, and stress reduction. These programs not only enhance physical flexibility but also contribute to mental well‐being, which is crucial for managing chronic pain conditions [10].

These sample exercises include seated hamstring stretches, standing quadriceps stretches, cat‐cow mobility exercises, and supine spinal twists. Stretching should be performed daily, holding each position for 20–30 s and avoiding any pain‐provocation. Regular practice of these exercises can lead to improved flexibility and reduced discomfort in elderly individuals with LBP [47].

### 3.4. Neuromuscular and Balance Exercises

Neuromuscular training targets proprioception, postural control, and dynamic balance. These capacities decline with age and are often impaired in individuals with chronic LBP, increasing fall risk and functional limitations. Therefore, balance exercises used to retrain spinal reflexes and motor control are often disrupted by persistent pain [48]. Yang et al. conducted a randomized trial evaluating Tai Chi for elderly adults with chronic LBP. Moreover, its key outcomes included a 30% reduction in average pain intensity, improved balance confidence measured by the Activities‐specific Balance Confidence Scale, and fewer reported falls over 6 months [49].

## 4. Mind‐Body Exercises (Yoga, Tai Chi, Qigong)

Mind‐body modalities, such as yoga, qigong, and tai chi, integrate physical movement, breath control, mindfulness, and relaxation techniques [50]. These practices are particularly beneficial for elderly individuals with LBP, as they address both physical and psychological aspects of pain. They not only alleviate physical stiffness and weakness but also mitigate psychological contributors to pain and contribute to the body′s self‐healing ability [51]. Recent clinical studies have demonstrated the effectiveness of mind‐body interventions in older populations with chronic LBP. In a RCT, Groessl et al. investigated a 12‐week yoga program among older U.S. military veterans with chronic LBP. Participants reported significant reductions in both average and worst pain intensity scores after the intervention [10]. The same study also observed improvements in mood, sleep quality, and overall satisfaction with life and physical functioning, highlighting the holistic benefits of yoga for elderly patients with chronic pain [10].

### 4.1. Aquatic Exercise (Hydrotherapy)

Water‐based exercises provide a unique and effective modality for managing chronic LBP in elderly individuals, offering both physical and psychological benefits [52]. The buoyancy of water reduces the load on joints, making movement easier and less painful for those with joint degeneration or obesity. Additionally, the natural resistance of water allows for muscle strengthening without high‐impact stress, whereas the warm temperature commonly used in therapeutic pools aids in relaxation and improved circulation [52, 53]. A 2021 meta‐analysis conducted by Faíl et al. assessed the effectiveness of aquatic therapy in elderly individuals with chronic LBP and other musculoskeletal diseases. The study found significant improvements in pain reduction, walking ability, and lumbar range of motion. Importantly, aquatic interventions also had a lower dropout rate, likely due to increased comfort and reduced pain during sessions. Furthermore, maintaining water temperature between 37°C and 38°C was shown to enhance therapeutic outcomes by promoting muscle relaxation and peripheral blood flow, which is especially beneficial for elderly patients with chronic pain [52].

When prescribing water‐based therapy for elderly individuals, sessions should ideally be scheduled two times per week, with each lasting 40 min [54]. The use of flotation devices, handrails, and pool lifts enhances safety and accessibility. Additionally, because elderly individuals may have underlying cardiovascular conditions, continuous monitoring of heart rate and perceived exertion is essential during sessions to ensure tolerance and avoid complications [55].

### 4.2. Barriers to Physical Activity and Solutions

Despite the well‐known benefits of physical activity, many elderly individuals with LBP remain inactive due to multiple physical challenges. Pain, fatigue, and fear of injury are commonly reported, often discouraging initiation or continuation of exercise programs [4]. Moreover, comorbidities such as osteoarthritis and cardiovascular disease further increase the risk perception around physical activity in this age group [56]. Additionally, psychological barriers, including depression, low motivation, and fear‐avoidance beliefs, are also highly prevalent in older adults with chronic pain [57].

Moreover, the lack of access to safe and accessible exercise environments especially in low‐income or rural areas remains a significant structural barrier to regular physical activity [58]. The COVID‐19 pandemic also exacerbated these barriers, leading to increased social isolation and reduced participation in physical programs [59]. Although within the healthcare system, time constraints and risk aversion among clinicians often result in the under‐prescription of exercise for elderly patients with LBP [60].

The medical model′s focus on pharmacologic rather than behavioral interventions further limits integration of movement‐based care. Lastly, evidence suggests that social support through peer groups, buddy systems, or caregiver involvement significantly increases adherence to physical activity among older adults [61, 62]. Programs that highlight perceived benefits such as improved sleep, reduced pain, and enhanced mobility tend to be more successful in maintaining participation [63, 64]. Structured routines, such as scheduled sessions with set goals and reinforcement from trusted healthcare providers, improve long‐term compliance [13].

### 4.3. Conflicting Evidence and Research Gaps

Although the benefits of physical activity for chronic LBP in older adults are well recognized, research findings are not always consistent [38]. For instance, a 2015 meta‐analysis by Searle et al. found no consistent superiority of one exercise modality over another, highlighting that patient preference and adherence may be more critical than the specific intervention itself [65]. Mind‐body practices like yoga and qigong often yield delayed improvements in function and pain perception, which may not be captured in short‐duration trials [49]. Furthermore, variability in study designs, outcome measures, and participant characteristics continues to hinder direct comparisons across trials.

A major limitation in current research is the frequent reliance on small sample sizes, especially when studying the oldest old or those with cognitive impairments. Moreover, many available trials have follow‐up periods of less than 12 months, failing to capture long‐term maintenance of benefits or recurrence of symptoms. Importantly, frail or multimorbid older adults who are common in clinical settings are often excluded from trials, thereby limiting external validity and real‐world applicability [66]. Additionally, combining physical activity with behavioral support or education in multimodal programs may better reflect clinical needs and improve adherence [67, 68]. Overall, a proper implementation of science frameworks is needed to identify scalable strategies for embedding successful programs into routine care [69–72].

## 5. Conclusions

In conclusion, LBP remains a prevalent and debilitating condition among the elderly population, significantly affecting their quality of life and independence. This review highlights that physical activity, particularly tailored exercise interventions, plays a vital role in both the prevention and management of LBP in older adults. Regular engagement in aerobic, strengthening, stretching, and balance exercises has been associated with reduced pain intensity, improved physical function, and enhanced psychological well‐being. Furthermore, maintaining an active lifestyle helps counteract age‐related musculoskeletal decline, thereby mitigating the progression of LBP symptoms. As the aging population continues to grow, the implementation of community‐based physical activity programs becomes increasingly essential to support elderly individuals in maintaining mobility and reducing the risk of chronic back pain.

Additionally, multidisciplinary approaches that combine physical activity with education, psychological support, and individualized care plans have shown promise in producing long‐term benefits for older adults with LBP. Considering the physical constraints and comorbidities of their older patients with LBP, healthcare providers should undertake early evaluations and encourage exercise adherence. This method is in line with the rising body of research that shows regular physical exercise is an effective, low‐risk strategy for reducing the symptoms of LBP and other age‐related health problems, as well as for improving overall quality of life. Research on exercise regimens and methods for encouraging older persons to stick to them should continue. Future research should aimed at identifying the most effective exercise protocols and strategies for enhancing long‐term compliance among older adults. Overall, physical activity represents a safe, cost‐effective, and impactful intervention that not only alleviates LBP but also fosters healthier aging and improved quality of life for elderly populations, as supported by the evidence presented throughout this review. Physical activity should be the initial line of treatment for lumbar pain in the elderly, and multicomponent programs that incorporate all three of these mechanisms have been shown to be more effective than unimodal approaches.

## Funding

This study was supported by Prince Sattam bin Abdulaziz University (PSAU/2024/R/1446).

## Conflicts of Interest

The authors declare no conflicts of interest.

## Data Availability

Data sharing is not applicable to this article as no datasets were generated or analyzed during the current study.
